# P-1793. Revitalizing Antibiotic Selection Through De-labeling of Outdated Antibiotic Allergies: Insights to Improving Antimicrobial Stewardship from South India

**DOI:** 10.1093/ofid/ofae631.1956

**Published:** 2025-01-29

**Authors:** Jasper Victoria Leelarani Martinraj, Sureshkumar Dorairajan, Surendhar Amargeeth, P Pooja, J Shanmugam

**Affiliations:** C. L. Baid Metha College of Pharmacy, Chennai, Tamil Nadu, India; Best of IDs, Chennai, Tamil Nadu, India; VISTAS, Chennai, Tamil Nadu, India; Madras Medical Foundation, Chennai, Tamil Nadu, India; Madras Medical Foundation, Chennai, Tamil Nadu, India

## Abstract

**Background:**

Self-reported drug allergies need to be revised or re-assessed, often. Therefore, this study aims to assess self-reported drug allergies among patients and identify potential candidates for de-labeling outdated or inappropriate antibiotic allergies, as part of antimicrobial stewardship interventions promoting rational antibiotic prescribing, as they facilitate the use of overly-broad spectrum antibiotics contributing to increased risk of antimicrobial resistance and adverse effects.

**Table 1:**
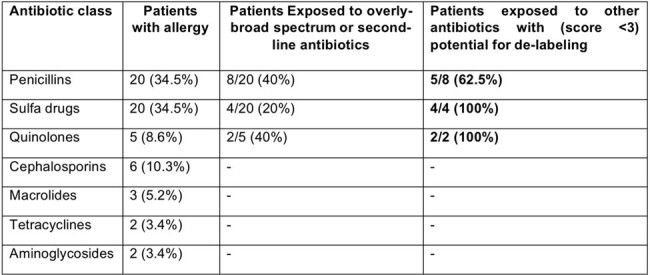
Antibiotic allergies and Potential candidates for de-labeling in the study

**Methods:**

This observational study was conducted in three tertiary care hospitals in South India from October to November 2023. Patients with drug allergy labels underwent thorough interviews utilizing the PEN-FAST clinical decision rule, adopted for risk stratification of all antibiotic allergies. The rule employed a score < 3 to identify low-risk penicillin allergies, potentially eligible for allergy de-labeling. Self-reported drug allergies were documented in an Excel sheet, with descriptive statistics were applied for analysis.

**Results:**

Among 857 patients analyzed, 58 (74.3%) patients reported antibiotic allergies. Penicillin and Sulfa allergy labels were the most prevalent allergy labels. Notably, 62.5% patients with low-risk Penicillin allergy and a vast majority of patients with Sulfa and Quinolone allergy are potential candidates for de-labeling, after employing the validated tool assessing the timeframe, severity and characterization of allergic reactions.

**Conclusion:**

In summary, The results of the study underscores the importance of adopting validated tools to revitalize antibiotic selection, de-labeling of > 60% inappropriate or outdated allergies and hence, enabling the use of first-line antibiotics in approximately 50-100% of the patients labeled with antibiotic allergies.

**Disclosures:**

**All Authors**: No reported disclosures

